# The NSP14/NSP10 RNA repair complex as a Pan-coronavirus therapeutic target

**DOI:** 10.1038/s41418-021-00900-1

**Published:** 2021-12-03

**Authors:** Gergely Rona, Andras Zeke, Bearach Miwatani-Minter, Maren de Vries, Ramanjit Kaur, Austin Schinlever, Sheena Faye Garcia, Hailey V. Goldberg, Hui Wang, Thomas R. Hinds, Fabrice Bailly, Ning Zheng, Philippe Cotelle, Didier Desmaële, Nathaniel R. Landau, Meike Dittmann, Michele Pagano

**Affiliations:** 1 Department of Biochemistry and Molecular Pharmacology, NYU Grossman School of Medicine, New York, NY 10016 USA; 2 Laura and Isaac Perlmutter NYU Cancer Center and NYU Grossman School of Medicine, New York, NY 10016 USA; 3 Howard Hughes Medical Institute, NYU Grossman School of Medicine, New York, NY 10016 USA; 4 Institute of Enzymology, Research Centre for Natural Sciences, Budapest, HU-1117 Hungary; 5 Department of Microbiology, NYU Grossman School of Medicine, New York, NY 10016 USA; 6 Department of Pharmacology and University of Washington, Seattle, WA 98195 USA; 7 Howard Hughes Medical Institute, University of Washington, Seattle, WA 98195 USA; 8 Univ Lille, INSERM, CHU Lille, UMR-S 1172, Lille Neuroscience and Cognition Research Center, F-59000 Lille, France; 9 ENSCL-Centrale Lille, CS 90108, F-59652 Villeneuve d’Ascq, France; 10 Institut Galien, Université Paris-Saclay, 92296 Châtenay-Malabry, France

**Keywords:** DNA repair enzymes, Microbiology, Infectious diseases

## Abstract

The risk of zoonotic coronavirus spillover into the human population, as highlighted by the SARS-CoV-2 pandemic, demands the development of pan-coronavirus antivirals. The efficacy of existing antiviral ribonucleoside/ribonucleotide analogs, such as remdesivir, is decreased by the viral proofreading exonuclease NSP14-NSP10 complex. Here, using a novel assay and in silico modeling and screening, we identified NSP14-NSP10 inhibitors that increase remdesivir’s potency. A model compound, sofalcone, both inhibits the exonuclease activity of SARS-CoV-2, SARS-CoV, and MERS-CoV in vitro, and synergistically enhances the antiviral effect of remdesivir, suppressing the replication of SARS-CoV-2 and the related human coronavirus OC43. The validation of top hits from our primary screenings using cellular systems provides proof-of-concept for the NSP14 complex as a therapeutic target.

## Introduction

Coronaviruses (CoVs) are positive strand RNA viruses with a replication property unique for RNA viruses - proofreading, executed by a viral exonuclease (ExoN). The proofreading function, which depends on the ExoN activity, allows for maintenance of the large CoV RNA genome by decreasing the mutation rate of the error-prone viral RNA-dependent RNA polymerase. ExoN activity is conferred by the NSP14-NSP10 complex (NSP14/10), in which NSP14, a bifunctional dsRNA exonuclease/guanosine 5ʹ methyltransferase protein, acts as the catalytic subunit activated by NSP10. The sequence and structure of NSP14 ExoN domain is highly divergent from most cellular exonuclease enzymes, including its closest mammalian relative TREX1, a DNA exonuclease. This disparity makes NSP14/10 an ideal anti-viral drug target. Importantly, the ExoN catalytic site and its surroundings are highly conserved across most CoVs (Supplementary Fig. 1A, B), suggesting the potential for pan-CoV inhibition. The ExoN domain of NSP14 resembles that of a eukaryotic DEDD exonuclease, which, to carry out its nucleotide excision, relies on negatively charged amino acids (in general, three aspartic acids and one glutamic acid) and bivalent metal ions. Specifically, NSP14 has been shown to rely on Mg^2+^ for its catalytic activity [1–3]. Mutations in NSP14 that impair proofreading function have been shown to critically reduce viral fitness [4–8]. Therefore, inhibition of ExoN is expected to be detrimental for CoV replication. In addition, based on genetic studies ExoN inhibition is expected to increase CoV sensitivity to chain terminating and mutagenic nucleotides [6, 9–11]. Using a novel, FRET-based exonuclease assay, we have screened small molecule compounds to identify NSP14/10 inhibitors. We then tested the antiviral potency of our best hits using viral replication assays with HCoV-OC43 and SARS-CoV-2. There results of these studies is presented herein.

## Results

**Fig. 1:**
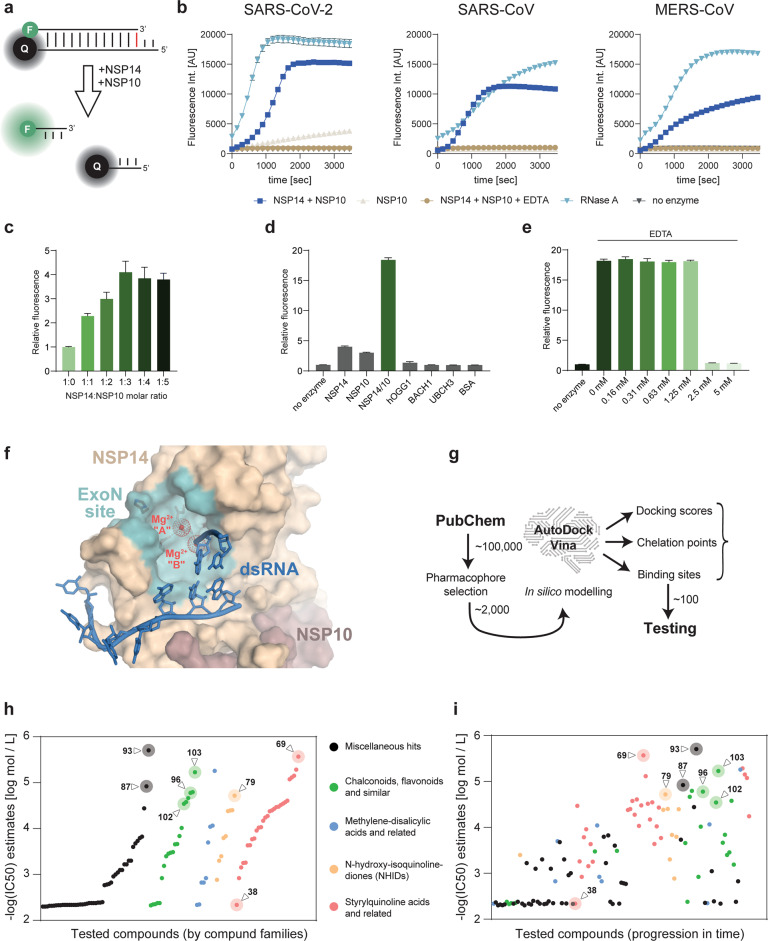
In vitro NSP14/10 activity assay and inhibitor screening. **a** Schematics showing dsRNA FRET-based NSP14 exonuclease activity assay. NSP14/10 recognizes the terminal mismatch and removes bases from the 3ʹ end of the substrate strand until the two RNA strands separate and the fluorescence signal increases. **b** Activity curves of SARS-CoV-2, SARS-CoV and MERS-CoV NSP14/10 complex using Oligo D. RNase A was used as a positive control and NSP10 alone was used as a negative control. For all downstream assays SARS-CoV-2 NSP14/10 was used in combination with Oligo D unless otherwise stated. Data shown from one independent measurement using technical duplicates. Error bars represent SD. **c** Bar graph shows exonuclease activity of the NSP14:NSP10 complex at different molar ratios. Relative activity was normalized to NSP14 alone. Fluorescence was read after 1 h of reaction time. **d** Specificity of the NSP14/10 complex over a variety of purified proteins (8-Oxoguanine DNA Glycosylase [OGG1]; BTB Domain and CNC Homolog 1 [Bach1]; Ubiquitin conjugating enzyme UbcH3; bovine serum albumin [BSA]). As proposed by the literature, NSP14 alone barely shows activity. Relative activity was normalized to a reaction mixture without any added purified protein. Fluorescence was read after 1 h of reaction time. **e** Bar graph shows EDTA sensitivity of the NSP14/10 exonuclease activity. Relative activity was normalized to a reaction mixture without NSP14/10. Fluorescence was read after 1 h of reaction time. **f** Structural model used for the in silico screening showing the NSP14 ExoN domain site with Mg^2+^ ions (red) and substrate dsRNA (blue). Surface of the ExoN catalytic site is colored light blue. Core catalytic residues are shown in stick representation. **g** Schematics of our computer-assisted drug design (CADD) and heuristic in silico screening approach. **h** 122 compounds and their -log(IC_50_) values (either exact or extrapolated) against NSP14/10 activity. Compounds listed on the X-axis are grouped and colored coded according to their various chemical families (left graph). **i** 122 compounds and their -log(IC_50_) values (either exact or extrapolated) against NSP14/10 activity. Compounds listed on the X-axis are ordered in progression over time (X-axis) and colored coded according to their various chemical families (right graph). Inhibitors were discovered using an iterative computer-assisted drug design and testing approach, leading to progressively better compounds over time.

The in vitro catalytic activity of NSP14/10 was previously mostly detected in gel-based assays, which are not amenable for high-throughput screenings [1, 3, 12]. We designed a novel, FRET-based exonuclease assay that allows high throughput assessment of potential NSP14/10 inhibitory compounds. We used dsRNA probes with sufficiently low Tm, labeled with a fluorophore and a quencher at a physical proximity (Fig. 1A, Supplementary Fig. 2A, B, and Table S1). NSP14/10 recognizes the terminal mismatch and removes bases from the 3ʹ end of the substrate strand until the two RNA strands separate, and the fluorescence signal increases. This assay provides a more accessible alternative to the recently described mass-spectrometry based screening method [13]. Using purified, recombinant NSP14/10 from either SARS-CoV-2, SARS-CoV, or MERS-CoV (Supplementary Fig. 2A), the reaction reached completion within 25 min (Fig. 1B and Supplementary Fig. 2B). All NSP14/10 complexes showed similar activity on the FRET dsRNA probes. In agreement with the literature, an excess of NSP10 helps NSP14 activity (Fig. 1C). So, for all downstream assays, NSP14/10 was used at a 1:3 molar ratio unless otherwise stated. The specificity of the reaction was demonstrated by the lack of signal in samples containing either NSP10 alone, NSP14/10 in the presence of EDTA (a Mg^2+^ chelating agent that inhibit the ExoN activity), or unrelated purified proteins (Fig. 1B and D). NSP14/10 exonuclease was inhibited by EDTA at a concentration of ~2.5 mM, which can chelate free Mg^2+^ present in the reaction mixture at 2 mM concentration (Fig. 1E).

**Fig. 2:**
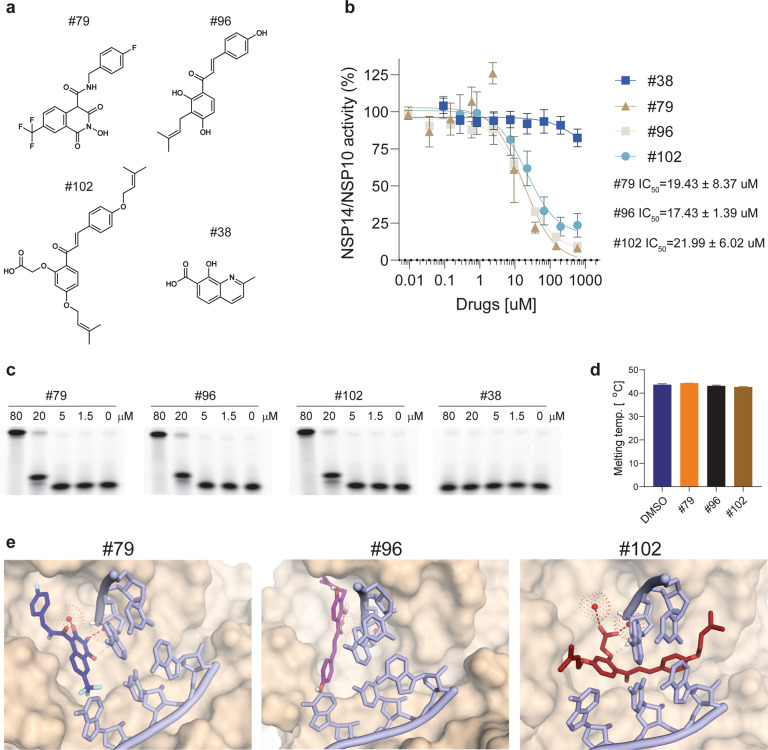
Characterization of novel NSP14/10 inhibitors. **a** Chemical structures of the three compounds (#79, #96 and #102) that performed the best in the viral infection assays. Compound #38, which does not inhibit NSP14, was included in downstream assays as a negative control. **b** Dose-response curves of NSP14/10 activity in the presence of compounds #38 (negative control), #79, #96 and #102. Average IC_50_ values were calculated from three independent measurements using technical duplicates. Error bars represent SD. **c** A gel-based assay was used to verify inhibitory potential of compounds #79, #96, #102, and #38. Inhibition of the NSP14/10 exonuclease activity results in the reduction of the full-length dsRNA oligo and increase in faster migrating bands. Compounds were used at the indicated concentrations. **d** Differential scanning fluorimetry performed in the presence or absence of the indicated compounds at 50 μM concentrations. Graph shows calculated mean melting temperatures of the NSP14/10 complex in the presence of the indicated compounds. Error bars represent SD. **e** Docking poses of compounds, predicted to stack either against the last nucleobase (#79, #96) or under the RNA (#102) while simultaneously engaging the Mg^2+^ ion(s) at the catalytic site (red dots).

The available structures of NSP14/10 of both SARS-CoV-2 and SARS-CoV display a canonical DEDDh ExoN catalytic site, which coordinates Mg^2+^ ions [2, 12, 14, 15]. We first selected 25 compounds that are either broad-spectrum nuclease inhibitors or FDA-approved Mg^2+^ chelator drugs. A number of compounds including Dolutegravir, Dicoumarol, *N-*Hydroxy-isoquinoline-1,3-dione and, 5,5ʹ-Methylenedisalicylic acid, showed detectable NSP14/10-inhibitory activities using our FRET assay. The results of these initial tests were instrumental to develop structural models of the NSP14 ExoN catalytic site that includes two Mg^2+^ ions, a substrate nucleic acid, and individual active compounds. This modeling incorporates 3 main principles: (i) both Mg^2+^ ions are appropriately coordinated by the enzyme and chelated by the inhibitor; (ii) NSP14 is loaded with an RNA substrate in its post-hydrolysis state; and (iii) the highly conserved ExoN site normally occupied by the last 3ʹ nucleotide is free to be occupied by the inhibitors stacking against the rest of the RNA (Fig. 1F). Using these criteria, we filtered ~100,000 compounds and subsequently narrowed them down to a list of ~2000 compounds that are structurally compatible to the active site. These compounds were then subjected to in silico docking and scored for docking energy, chelation potential, and the possibility to bind the ExoN site (Fig. 1G). Based on the docking scores, we picked 122 compounds for in vitro testing using our FRET-based assay, which led to the identification of 23 inhibitors with an IC_50_ lower than 40 μM (Fig. 1H, I, Supplementary Fig. 3, Supplementary Fig. 4, and Table S2). In addition to various isolated hits, we identified 4 families of compounds as NSP14 inhibitors: (1) Methylenedisalicylic acids, (2) *N*-hydroxy-isoquinoline-1,3-diones, (3) 2-Styryl-quinoline derivatives, and (4) Chalconoids. Figure 1H shows the compounds grouped by families and potency, while Fig. 1I shows the progression in identifying better compounds over time, providing a validation of our iterative computer-assisted drug design and testing approach. Importantly, the low μM IC_50_ compounds (#69, #79, #87, #93, #96, #102, #103) were similarly potent in inhibiting the NSP14/10 complex of both SARS-CoV and MERS-CoV (Fig. 2A, B, Supplementary Figs. 3, 4, and Table S2). While EDTA was only able to inhibit NSP14 activity when its concentration exceeded that of the free Mg^2+^ (Fig. 1E), the inhibitors identified here, worked at concentrations below that of free Mg^2+^, arguing for their ability to chelate the Mg^2+^ present in the catalytic pocket. We also confirmed the inhibitory potential of select compounds using gel electrophoresis as an orthogonal method (Fig. 2C and Supplementary Fig. 5A). Differential scanning fluorimetry proved that none of the tested inhibitors affected the melting temperature of the complex, suggesting that they do not work by affecting the folding of the NSP14/10 complex (Fig. 2D and Supplementary Fig. 5B). Finally, none of the tested compounds showed quenching or autofluorescence, excluding false positive and false negative hits (Supplementary Fig. 5C). Docking of our best candidates into a post-catalytic model of the NSP14-NSP10-dsRNA complex shows shared as well as unique features. One common theme is that the compounds stack against the penultimate nucleobase with a planar, aromatic ring system and coordinate the Mg^2+^ ions using one or more substituents. The most probable docking poses of compounds #79 and #96 (Fig. 1F and Fig. 2E) showed a largely overlapping binding site focused at Mg^2+^ “A” (the catalytic metal ion). They occupy the site of the last, cleaved nucleotide while also extending towards the mobile, catalytic His (especially with compound #79). Compound #102 likely binds in a different fashion, adopting a pose with a weaker stacking against the penultimate nucleotide and sandwiching in-between the incoming RNA strand and the protein surface instead (Fig. 2E).

**Fig. 3:**
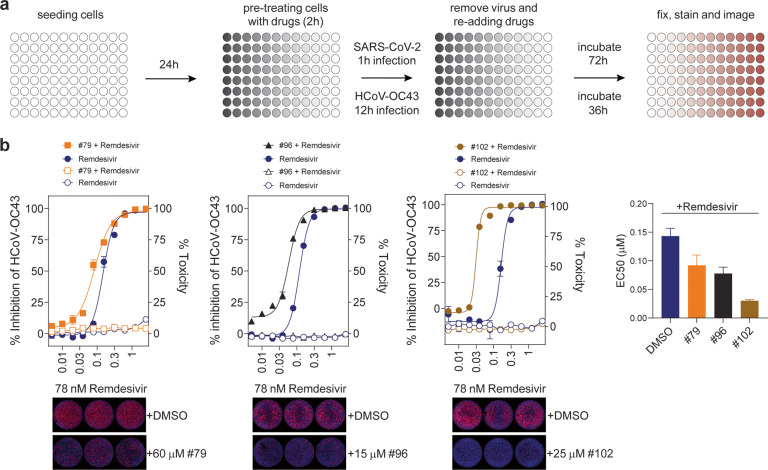
Synergistic effect of select compounds with remdesivir using HCoV-OC43 viral infection assay. **a** Schematics showing the viral infection assay workflow. Drug treated cells were infected with HCoV-OC43 or SARS-CoV-2, fixed, stained, and imaged at indicated times post infection. Cytotoxicity was measured in similarly drug-treated uninfected cells using either the CellTiter-Glo or the alamarBlue assay. **b** Representative graphs are shown of the antiviral activity (full symbols) and cytotoxicity (empty symbols) of compounds #79, #96 and #102 in HCM3 cells infected with HCoV-OC43. Compounds were applied at the following concentrations: #79: 60 μM, #96: 15 μM, #102: 25 μM. Remdesivir concentrations are indicated on the X-axis in μM. Error bars represents SEM. IF images of representative wells show anti-N staining (red) and DAPI signal (blue) at indicated drug concentrations. Graph shows the EC_50_ values from three independent experiments using technical triplicates. Error bars represents SEM.

**Fig. 4:**
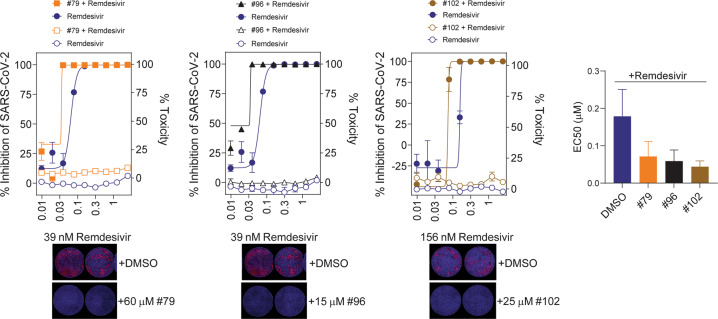
Synergistic effect of select compounds with remdesivir using SARS-CoV-2 viral infection assay. Representative graphs are shown of the antiviral activity (full symbols) and cytotoxicity (empty symbols) of compounds #79, #96 and #102 in A549^+ACE2^ cells infected with SARS-CoV-2. Compounds were applied at the following concentrations: #79: 60 μM, #96: 15 μM, #102: 25 μM. Remdesivir concentrations are indicated on the X-axis in μM. Error bars represents SEM. IF images of representative wells show anti-N staining (red) and DAPI signal (blue) at indicated drug concentrations. Graph shows the EC_50_ values from three independent experiments using technical triplicates. Error bars represents SEM.

Next, we sought to test our compounds in viral infection assays. To this end, compounds displaying low μM IC_50_ (#69, #79, #87, #93, #96, #102, #103) were tested individually or in combination with remdesivir for their ability to inhibit the infection by the human seasonal CoV, HCoV-OC43, and the pandemic CoV, SARS-CoV-2. To distinguish a direct effect on viral replication from an effect on the viability of the host cells, we in parallel measured the cellular toxicity of all compounds in uninfected cells. As the HCoV-OC43 ExoN domain shows high sequence similarity to that of SARS-CoV2 (Supplementary Fig. 1A), the use of HCoV-OC43 allowed us to screen the top hit compounds in a BSL2 environment to evaluate pan-CoV activity. Viral spread in the presence of serial compound dilutions was evaluated by high-content imaging and analysis as previously described (Fig. 3A) [16]. None of the compounds had detectable inhibitory activity against HCoV-OC43 on their own (Supplementary Fig. 6A, B); however, they synergized with remdesivir (Fig. 3B and Supplementary Fig. 7A-B). Compounds #79 (7-trifluoromethyl-N-(4-fluorobenzyl)−2-hydroxy-1,3-dioxo-4H-isoquinoline-4-carboxamide) [17], #96 (Isobavachalcone), and #102 (Sofalcone) showed the highest synergistic effect with remdesivir, lowering the EC_50_ values of remdesivir by ~5-fold. The results with SARS-CoV-2, performed in a BSL3 environment, closely mirrored the HCoV-OC43 results. While no direct antiviral effect was detected for the drugs on their own (#69, #79, #87, #93, #96, #102, #103) (Supplementary Fig. 8A-B), compounds #79, #96 and #102 synergized with remdesivir (Fig. 4 and Supplementary Fig. 9A-B). This synergism is substantial considering the rather high individual IC_50_ of these compounds (~30 μM).

## Discussion

By combining a novel assay measuring the catalytic activity of NSP14/10 with in silico modeling and screening, we identified a series of inhibitors of the coronavirus NSP14/NSP10 enzyme complex showing activity both in vitro and in vivo (Supplementary Fig. 9C). CoVs rely on their ExoN activity for proper propagation [4, 5], which is evident when measured over many replication cycles. The temporal limitation of our assay only allows up to a maximum of 2-3 replication cycles, which may explain why our inhibitors do not show activity on their own. In contrast, the inhibitors display sensitization of viral replication to remdesivir, providing an important proof of concept for a new therapeutic approach against CoVs. Ribonucleotide analogs working as chain terminators do not induce lethal mutagenesis and can be subdivided into direct or delayed terminators. Direct chain terminators are incorporated at the end of RNA strand without being followed by other ribonucleotides. Therefore, they are immediately sensed by the RNA-dependent RNA polymerase (NSP12) and by NSP14, and can be removed from the viral genome in a single step. Remdesivir’s incorporation is instead followed by ~ 3 ribonucleotides, acting as a delayed chain terminator [18–20]. We believe that remdesivir incorporated into nascent RNA is an excellent target for NSP14 ExoN inhibitory drugs since multiple ribonucleotides need to be removed until remdesivir can be excised from the viral genome [21]. This provides a longer window of opportunity for the inhibitors to act on NSP14.

**Fig. 5:**
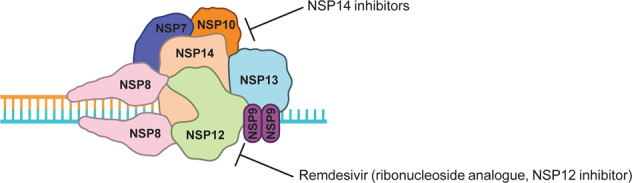
NSP14/10 inhibition as a therapeutic approach. Schematics showing our therapeutic approach where inhibiting NSP14 potentiates the inhibition of NSP12 (with remdesivir, a ribonucleoside analog).

When evaluating our compounds, one must note that the degree of antiviral efficacy and synergy with remdesivir is subject to a multitude of biological factors influencing drug uptake and availability. Of our top hits displaying synergy with remdesivir, isobavachalcone (#96) is extracted from medicinal herbs [22]; compound #79 was originally synthesized as an early-stage anti-HIV agent [23]; and sofalcone, albeit not FDA-approved, has been demonstrated by clinical studies in Japan to be well tolerated by humans for the treatment of gastric diseases [24, 25]. Optimization of these hits has the potential to develop them into potent pan-CoV therapeutics (Fig. 5).

## Materials and methods

### Development of the dsRNA-loaded NSP10-NSP14 model

Based on the nearly 100% sequence homology around the ExoN catalytic site of SARS-CoV-2 and SARS-CoV, we used SARS-CoV NSP14 crystal structures in our modeling. Several published structures are available (5C8T, 5C8U and 5C8S, with one Mg^2+^ ion (Mg^2+^ “B”) at the catalytic site). The structure, 5NFY, was taken as our starting template, due to its decent electron density map. Although structure 5NFY contains no explicit Mg^2+^ ions, the octahedral coordination of a single density near the catalytic site (H_2_O in the PDB file) allowed its re-interpretation as the metal ion Mg^2+^ “B”. The predicted site of the other catalytic metal ion, Mg^2+^ “A” contained no matching electron density in the published NSP14 structures and was therefore determined to be a highly conserved metal ion binding site which is only occupied in the full, substrate-bound enzyme. Structures were adjusted using Coot (version 0.8.6.1) and PyMol (version 1.8), and protonated on the MolProbity webserver (including His, Gln and Asn ring flips). The protein models were prepared for docking using AutoDockTools (version 1.5.7), removing Zn^2+^ ions, merging non-polar hydrogens and adding Kollman charges. Mg^2+^ ions, that have no default charge under the Kollman system, were manually assigned a charge +2.0. We did not to use fractional charges, to offset the lack of chelation energies under AutoDock. Although derived from SARS-CoV, the final model also matched well with published SARS-CoV-2 NSP14 structures (PDB entries 7MC5, 7MC6 and 7DIY).

To model the RNA-bound NSP14-NSP10 complex, we utilized the structure of the very distantly homologous TREX1 as a guide, loaded with dsDNA (PDB: 4YNQ). The position of the substrate in this model is also very similar to other DEDD exonucleases—such as human ERI1 (PDB: 4QOZ), yeast Pan2 (PDB: 6R9J) exonucleases or the Lassa virus nucleoprotein (PDB: 4FVU). The dsRNA in our model was adjusted to maximize H-bonding with the catalytic site of ExoN (using PyMol and Coot), and the last nucleotide positioned into the catalytic site, with Mg^2+^ ions at a canonical phosphate coordinating position. The RNA strands were also intentionally slightly distorted (in Coot), to mimic a mismatching 3ʹ end. Our resulting model was very similar to those determined experimentally later, including the apo NSP14 (PDB: 7MC6) and substrate-loaded NSP14 complexes (PDB: 7N0B). Then the last nucleotide was removed from the final complex (with its phosphate). We argued that any compound that could stabilize this post-catalytic state (that is also pre-catalytic if the dsRNA does slide into place with one position off) would be an effective inhibitor. Then we prepared this model for docking using MolProbity, PyMol and AutoDockTools as outlined previously. Compounds docking into this model with high scores usually show at least some inhibition in our assay, confirming our approach.

### Selection of candidate compounds

The very initial set of compounds was selected from a pool of pan-enzyme inhibitors and FDA-approved drugs known to act on viral endonucleases, exonucleases or integrases, without any docking simulations. Out of this pool only a few candidates showed inhibitory potency. Because these molecules can adopt a pose that avoids half of the catalytic site (where the RNA is proposed to enter), we concluded that the RNA must be present in the model to yield meaningful results. In the subsequent rounds, we utilized the fully dsRNA-loaded (post-catalytic) complex to successfully identify several potent inhibitors. Candidates for docking were selected from publicly available and commercial databases (PubChem, ZINC, Mcule, MolPort, ChemFaces, etc.) containing “drug-like” candidates, with sufficient structural diversity. Once the very first hits were uncovered, we analyzed their structures and used them in subsequent database queries. In addition, new heuristically designed molecular architectures were introduced at each run to ensure scaffold diversity. We performed this method iteratively, until it converged on several potently inhibiting compound families.

### Docking of ligands

The computationally predicted 3D structure of candidate compounds was downloaded from PubChem. Whenever no structure was available in PubChem, we used either the OpenBabel or MolView websites to generate 3D molecular coordinates in a suitable format. Structures were then individually reviewed in PyMol, setting the predicted protonation state at pH = 7.0 and correcting their geometries when necessary. Reviewed molecules were converted into pdbqt files using AutoDockTools (adding Gasteiger charges). Special care was taken when setting the torsion tree in case of compounds with conjugated double bonds or aromatic systems, to avoid chemically implausible docking poses. Compounds were then docked into the NSP14/10 models using AutoDock Vina (version 1.1.2), with a fully rigid protocol, and a target box limited around the ExoN catalytic site (to save computation time and facilitate evaluation).

### Plasmid constructs

The cDNA encoding NSP14 and NSP10 of SARS-CoV-2, SARS-CoV, and MERS-CoV were codon optimized for expression in *E. coli* using the codon optimization tool of GenScript. Custom cDNAs were synthesized by GenScript and subcloned into a pET-30a(+) vector backbone, resulting in a C-terminal 6X His-tag following a flexible linker (GGGSGGGS) for all the constructs.

### Expression and purification of NSP14/10

NSP14 and NSP10 proteins of SARS-CoV-2, SARS-CoV and MERS-CoV were either purified in house or by GenScript. In brief, vectors encoding NSP14 and NSP10 were transformed into OverExpress C41(DE3) or BL21(DE3) *E. coli*. 5 L of *E. coli* were grown at 37 °C to an OD_600_ of 0.6, induced with 1 mM IPTG and shaken at 15 °C for 16 h. Cells were harvested and pelleted by centrifugation at 4000 x g for 30 min at 4 °C. Cell pellets were resuspended in lysis buffer (50 mM TRIS-HCl pH=8.0, 150 mM NaCl, 0.5% Triton X-100, 5 mM β-mercaptoethanol), lysed by sonication and clarified using centrifugation (30000 x g, 30 min at 4 °C). Clarified lysate was supplemented with 20 mM imidazole and incubated with Ni-NTA resin (Qiagen) 4 °C for 1 h, washed with wash buffer (50 mM TRIS-HCl pH=8.0, 300 mM NaCl, 5% glycerol, 40 mM imidazole and 5 mM β-mercaptoethanol), and eluted with elution buffer (50 mM TRIS-HCl pH=8.0, 100 mM NaCl, 350 mM imidazole, 5% glycerol and 5 mM β-mercaptoethanol). Eluate was dialyzed overnight into buffer without imidazole (50 mM TRIS-HCl pH=8.0, 150 mM NaCl, 5% glycerol and 1 mM DTT), and then sterilized by a 0.22 μm filter. Protein concentration was determined by Bradford protein assay with BSA as a standard and concentrated as necessary. Samples were then aliquoted and stored at −80 °C.

### FRET exonuclease activity assay

To prepare the dsRNA substrates, RNA oligos were annealed at a final concentration of 50 μM. To anneal the ssRNA oligos, samples were heated in a PCR cycler to 95 °C for 5 min and then cooled to 5 °C in 5 °C increments over 18 cycles of 1 min each. Exonuclease activity assays were performed at 37 °C in black bottom 96 well plates. The reactions were performed in the following buffer: 50 mM TRIS-HCl pH 7.5, 2 mM MgCl_2_, 2 mM DTT. NSP14 and NSP10 were used at 200 nM and 600 nM, respectively, maintaining a molar ratio of 1:3, while the dsRNA substrate was added at a final concentration of 1 μM. The fluorescence intensity of each well was measured every 150 s on an Infinite 200 PRO microplate reader (Tecan) over the course of the activity assay using the following settings: Excitation 490 nm (±9 nm)/ Emission 520 nm (±20 nm). Activity measurements for MERS-CoV were performed identically to SARS-CoV-2. Activity assays of SARS-CoV NSP14/NSP10 were performed at concentrations 40 nM and 120 nM, respectively.

### IC_50_ calculation

Selected inhibitors were serial diluted in the indicated range. The fluorescence of each dilution was measured in the linear activity range and used to determine the IC_50_ for selected compounds using the three-parameter non-linear regression function of GraphPad Prism (9.1.2). Activity of inhibited NSP14/10 was normalized to the control sample with the vehicle control.

### Compound screening

Screening was performed using a custom compound selection originating from commercial sources (Sigma, Selleck, MedChem Express, Tocris, Enamine, MCule, Key Organics, Hit2Lead, Vitas-M Laboratory and ChemFaces) as well as custom synthesized compounds from the Styrylquinoline and NHID groups, with each compound having an average purity of 95%. Compounds were reconstituted at 100 mM in DMSO, aliquoted and stored under argon gas at −80 °C. Primary screening was performed at 500 or 250 μM compound concentration, where compounds were pre-incubated with the NSP14/10 complex for 15 min at room temperature before adding the FRET substrate. Secondary screenings on select compounds were performed at 100 or 50 μM. To exclude false positive and negative hits, compounds at 250 μM were tested for autofluorescence and quenching. Compounds were color coded based on their chemical scaffold grouping. They were either ordered on the X-axis based on their chemical grouping (Fig. 1E) or in progression over time, (Supplementary Fig. 2F). For each compound tested, we plotted its -log (IC_50_) value on the Y-axis (either exact or extrapolated value).

### Exonuclease gel assay

6FAM labeled RNA oligos were annealed as described previously. The enzymatic reactions were performed as described in the FRET kinetic exonuclease activity assay but using 5 μM dsRNA substrate in a total volume of 20 μL. After 45 min, reactions were stopped with Novex™ Hi-Density TBE Sample Buffer containing EDTA (5 mM), and chilled on ice. 10 μL of samples were loaded into a 20% TBE Gel and run at 200 V for 80 min. Gels were imaged using a BIO-RAD Gel Doc™ XR + imager.

### Differential scanning fluorimetry

Melting temperature of the NSP14/10 complex in the presence or absence of select compounds were assayed by differential scanning fluorimetry using either Protein Thermal Shift™ (Thermo Scientific) or GloMelt™ Thermal Shift Protein Stability Kit (Biotium) according to the manufacturer’s protocol. Assays were performed in a 96-well qPCR plate in a final volume of 25 μL in the following reaction buffer: 50 mM TRIS-HCl pH=7.5, 2 mM MgCl_2_, 2 mM DTT. NSP14 and NSP10 were allowed to pre-complex at an equimolar ratio of 5 μM at RT for 15 min before adding the compounds at 50 μM final concentration. The temperature was linearly increased in a QuantStudio3 Real-Time PCR System with a step of 0.05 °C/s, from 25 °C to 95 °C, and fluorescence readings were taken at each interval (Protein Thermal Shift Assay: ROX filter set; GloMelt Thermal Shift Assay: FAM filter set). Melting temperatures were calculated as the inflection point of the melting curve using the derivative analysis function of the QuantStudio^TM^ Design & Analysis Software (version 1.4.3).

### Cell lines and viruses

Cell lines were purchased from ATCC. A549^+ACE2^ cells, were created as described previously [16]. A549^+ACE2^, HMC3 (ATCC CRL-3304) and MRC-5 (ATCC CCL-171) cells were maintained in Dulbecco’s modified Eagle medium (DMEM) supplemented with 10% fetal bovine serum (FBS), 1% penicillin/streptomycin and incubated at 37 °C under 5% CO_2_. Cell lines were routinely monitored for mycoplasma contamination using the Universal Mycoplasma Detection Kit (ATCC 30-1012 K). All cells used in this study tested negative for Mycoplasma contamination. A549^+ACE2^ cells were used for SARS-CoV-2 infection assays. SARS-CoV-2 isolate USA-WA1/2020 stocks were prepared as described previously [16]. HMC3 cells were used for HCoV-OC43 infection assays. HCoV-OC43 viral stocks (ATCC VR-1558) were propagated/isolated as detailed below. MRC-5 cells were seeded at a density of 2 × 10^6^ cells in 100 mm dish. The next day, the cells were infected with 3 × 10^6^ pfu/ml of HCoV-OC43 and incubated at 33 °C for four days until 90–100% cells have cytopathic effects. The culture supernatant and infected cells were harvested, centrifuged at 1000 × *g* for 5 min and the supernatant was stored at −80 °C.

### SARS-CoV-2 viral infectivity assay

A549^+ACE2^ cells were seeded into black 96-well plates at 90% confluency. The next day, media was removed and replaced with complete media containing compounds/carrier 2 h prior to infection. Cells were then infected to reach 80–90% infected cells after 72 h at 37 °C. One hour post virus addition, virus was removed, and media containing compounds/carrier was added. At 72 h post infection, cells were fixed by submerging in 10% formalin solution for 30–45 min. After fixation cells were washed once with H_2_O to remove excess formalin. Plates were dried and PBS was added to each well before exiting the BSL-3 facility. Fixed cells were permeabilized and stained with mouse monoclonal SARS-CoV anti-N antibody 1C7, which cross-reacts with SARS-CoV-2 N (kind gift of Thomas Moran), goat anti-mouse AlexaFluor 647 and DAPI. For determination of cytotoxicity, A549^+ACE2^ cells were seeded into opaque white wall 96-well plates. The following day, media was removed, replaced with media containing compounds/carrier and incubated for 72 h. At these timepoints, ATP levels were determined by CellTiter-Glo 2.0 (Promega) using a BioTek Synergy HTX multi-mode reader.

### HCoV-OC43 viral infectivity assay

HMC3 cells were seeded into black 96-well plates at 90% confluency. The next day, media was removed and replaced with complete media containing compounds/carrier 2 h prior to infection. After that, 5 μl of HCoV-OC43 virus was added to each well at an MOI of 0.005 for a final total volume of 100 μl/well. The plates were incubated at 33 °C. The viral suspension was removed after 12 h and drugs were added back to the cells to further incubate at 33 °C for 36 h. At 48 h post infection, cells were fixed with 4 % PFA in the media for 15 min. Residual PFA was quenched with a 50 mM NH_4_Cl and 100 mM NaCl solution. Cells were permeabilized and blocked in 5% FBS, 3% BSA, 0.05% Triton X-100 and 0.005% NaN_3_ in PBS for an hour. Anti-N antibody (Sigma 542-7D) was diluted in 4% FBS in PBS at 1:1000 dilution and was applied at 4 °C for 16 h. After several washing steps, cells were stained with AlexaFluor 594 and DAPI. For determination of cytotoxicity, HMC3 cells were seeded into opaque white wall 96-well plates. The following day, media was removed, replaced with media containing compounds/carrier and incubated for 48 h. After 48 h, cell viability was determined using alamarBlue™ Cell Viability Reagent using an Infinite 200 PRO microplate reader (Tecan).

### Imaging analysis

Plates were scanned on a Citation 5 Imaging Multi-Mode Reader (BioTek). Gen5 software (version 3.10) was used for both image acquisition and analysis. A total number of 16 images/well were collected at 4x magnification to span the entire well. DAPI channel was used to build primary masks for the nucleus. Secondary masks were built within a 15 μm ring around the primary nuclear mask. Infection efficiencies were calculated from the ratio of the total fluorescence intensity within the secondary mask and the total area of the secondary mask for each well. Microscope images were prepared for publication using ImageJ (version 1.53i).

### Antibodies

Antibodies used in the study were the following:

- SARS-CoV anti-N antibody 1C7, mouse monoclonal, cross-reacts with SARSCoV-2 N, kind gift of Thomas Moran, dilution: 1:1000

- HCoV-OC43 anti-N antibody, mouse monoclonal, Sigma-Aldrich, cat. no.: MAB9013, lot no.: 3587234, dilution: 1:1000

- Goat anti-Mouse IgG (H + L) Highly Cross-Adsorbed Secondary Antibody, Alexa Fluor Plus 594, cat. no.: A32742, lot no.: VL316327, dilution: 1:1000

- Goat anti-Mouse IgG (H + L) Highly Cross-Adsorbed Secondary Antibody, Alexa Fluor Plus 647, cat. no.: A32728, lot no.: VH311610, dilution: 1:1000

## Supplementary information

Supplementary Material

Supplementary Figure 1 Conserved nature of human coronavirus NSP14 proteins.

Supplementary Figure 2 <i>In vitro</i> FRET-based NSP14 activity assay optimization and characterization.

Supplementary Figure 3 IC<sub>50</sub> curves of select compounds.

Supplementary Figure 4 Activity curves of select compounds used to calculate IC<sub>50</sub> values.

Supplementary Figure 5 Confirmation of the inhibitors identified by our screening.

Supplementary Figure 6 Viral infection assay using HCoV-OC43.

Supplementary Figure 7 Synergistic effect of select compounds with remdesivir using HCoV-OC43 viral infection assay.

Supplementary Figure 8 Viral infection assay using SARS-CoV-2.

Supplementary Figure 9 Synergistic effect of select compounds with remdesivir using SARS-CoV-2 viral infection assay.

Reproducibility Checklist

Detailed Author Contribution Form

## Data Availability

All accession codes, unique identifiers, and web links for publicly available datasets are available within the article and the reporting summary. All original data are available from the corresponding authors upon request.
