# Supplementary material for: The NSP14/NSP10 RNA repair complex as a Pan-coronavirus therapeutic target
Source: Cell Death Differ. 2021 Dec 3;29(2):285–92. doi: 10.1038/s41418-021-00900-1 (PMC8640510; doi:10.1038/s41418-021-00900-1)
Supplement: Supplementary file 1 — Supplementary Material [file 41418_2021_900_MOESM1_ESM.docx]

**SUPPLEMENTARY MATERIAL**

**The NSP14/NSP10 RNA Repair Complex as a Pan-coronavirus Therapeutic Target**

Gergely Rona^†,1,2,3^, Andras Zeke^†,1,2,4^, Bearach Miwatani-Minter^1,2^, Maren de Vries^5^, Ramanjit Kaur^5^, Austin Schinlever^5^, Sheena Faye Garcia^1,2^, Hailey V. Goldberg^1,2^, Hui Wang^6,7^, Thomas R. Hinds^6,7^, Fabrice Bailly^8^, Ning Zheng^6,7^, Philippe Cotelle^8,9^, Didier Desmaële^10^, Nathaniel R. Landau^5^, Meike Dittmann*^,5^, Michele Pagano*^,1,2,3^

^1^Department of Biochemistry and Molecular Pharmacology, ^2^Laura and Isaac Perlmutter NYU Cancer Center and ^3^Howard Hughes Medical Institute, NYU Grossman School of Medicine, New York, NY 10016, USA

^4^Institute of Enzymology, Research Centre for Natural Sciences, HU-1117 Budapest, Hungary

^5^Department of Microbiology, NYU Grossman School of Medicine, New York, NY 10016, USA

^6^Department of Pharmacology and ^7^Howard Hughes Medical Institute, University of Washington, Seattle, WA 98195, USA

^8^Univ Lille, INSERM, CHU Lille, UMR-S 1172, Lille Neuroscience and Cognition Research Center, F-59000, Lille, France and ^9^ENSCL-Centrale Lille, CS 90108, F-59652 Villeneuve d’Ascq, France

^10^Institut Galien, Université Paris-Saclay, 92296 Châtenay-Malabry, France

^†^These authors contributed equally to this work.

*Correspondence: [michele.pagano@nyumc.org](mailto:michele.pagano@nyumc.org) and [meike.dittmann@nyumc.org](mailto:meike.dittmann@nyumc.org)

**SUPPLEMENTARY FIGURE LEGENDS**

**Supplementary Figure 1 Conserved nature of human coronavirus NSP14 proteins.**

**a,** Alignment of human pathogenic coronavirus NSP14 protein ExoN domains (including mild and severe disease causative agents). **b**, Structural model showing the conserved nature of the NSP14 ExoN catalytic site (with Mg^2+^ ions). The identical amino acids across a panel of coronaviruses are shown with stick representation. This figure was drawn by aligning the following *Coronaviridae* sequences (UniProt entries): P0DTD1 (SARS2), P0C6X7 (SARS), K9N7C7 (MERS), P0C6W6, P0C6W4, U5KNA9, P0C6W5, Q98VG9, P0C6X5, P0C6Y5, B2BW31, A3EXI9, A3EXH3, A3EXI1, I7AWB5, P0C6X8, P0C6X2, C6GHR8, P0C6W8, P0C6X6, P0C6X0, P0C6Y3, A0A0K2RW65, A0A4D6FWB1

**Supplementary Figure 2 *In vitro* FRET-based NSP14 activity assay optimization and characterization.**

**a,** Coomassie blue stained SDS-PAGE gel image of purified, recombinant NSP14 and NSP10 (5 μg / lane) used in downstream assays from SARS-CoV-2, SARS-CoV, and MERS-CoV. Asterisks denotes the band corresponding to NSP14 or NSP10. **b**, Several FRET-based dsRNA oligos were tested for optimal signal to noise ratio. Details of Oligo A-E are summarized in Table S1.

**Supplementary Figure 3 IC_50_ curves of select compounds.**

Dose-response curves of NSP14/10 activity in the presence of compounds #38 (negative control, with no observed inhibition), #69, #79, #87, #93, #96, #102 and #103. IC_50_ values were calculated from three independent experiments using technical duplicates for SARS-CoV-2 and from one experiment using technical duplicates for SARS-CoV and MERS-CoV. Error bars represent SD. Calculated IC_50_ vales are summarized in Table S2.

**Supplementary Figure 4 Activity curves of select compounds used to calculate IC_50_ values.**

Fluorescent intensity signal was plotted during the time course of NSP14/NSP10 inhibition across increasing concentrations of compounds (shown in μM on the right side on each graph) #69, #79, #87, #93, #96, #102, #103 and #38, as detected by FRET-based activity assay. The linear phase of these graphs, showing maximum initial velocity, were used to calculate the IC_50_ values shown in Supplementary Figure 2. Three independent experiments using technical duplicates for SARS-CoV-2 and one experiment using technical duplicates for SARS-CoV and MERS-CoV were carried out. Error bars represent SD.

**Supplementary Figure 5 Confirmation of the inhibitors identified by our screening.**

**a**, A gel-based assay was used to verify inhibitory potential of compounds #69, #87, #93 and #103. Inhibition of the NSP14/10 exonuclease activity results in the reduction of the full-length dsRNA oligo and increase in faster migrating bands. Compounds were used at the indicated concentrations **b,** Differential scanning fluorimetry performed in the presence or absence of the indicated compounds at 50 μM concentrations. Melting curves showing the means of the recorded fluorescence values are plotted in the upper graphs from one experiment using technical triplicates (AU, arbitrary units). The negative first derivative of the melting curves are plotted below to aid visualization. **c**, Compounds were tested against their ability to quench the fluorescent signal of 6FAM (resulting in false positive hits) and their autofluorescence (resulting in false negative hits) at the indicated wavelength at 250 μM final concentration. Graphs were normalized to DMSO. Error bars represent SD from one experiment using technical triplicates.

**Supplementary Figure 6 Viral infection assay using HCoV-OC43.**

**a,** Left graph shows anti-HCoV-OC43 activity of compounds #69, #79, #87, #93, #96, #102, #103 and remdesivir on their own, normalized to DMSO. Error bars represent SEM of one experiment from technical duplicates. Right graph shows cytotoxicity of compounds #69, #79, #87, #93, #96, #102, #103 and remdesivir on their own, normalized to DMSO. Error bars represents SEM of one experiment from technical duplicates. Compounds were applied in a 2-fold serial dilution across 4 concentrations. Starting drug concentrations for each compound were the following: #69: 6 μM, #79: 60 μM, #87: 30 μM, #93: 60 μM, #96: 15 μM, #102: 25 μM, #103: 3 μM and remdesivir: 5 μM. **b**, IF images show anti-N staining (red) and DAPI signal (blue) of the indicated compounds for the graphs above.

**Supplementary Figure 7 Synergistic effect of select compounds with remdesivir using HCoV-OC43 viral infection assay.**

**a**, Representative IF images showing anti-N staining (red) and DAPI signal (blue) of the experiment shown in Figure 3B. **b**, Graphs show two additional representative experiments of the antiviral activity (full symbols) and cytotoxicity (empty symbols) of compounds #79, #96 and #102 in HCM3 cells infected with HCoV-OC43 (done in technical triplicates). Compounds were applied at the following concentrations: #79: 60 μM, #96: 15 μM, #102: 25 μM. Remdesivir concentrations are indicated on the X-axis in μM. Error bars represent SEM.

**Supplementary Figure 8 Viral infection assay using SARS-CoV-2.**

**a,** Left graph shows anti-SARS-CoV-2 activity of compounds #69, #79, #87, #93, #96, #102, #103 and remdesivir on their own, normalized to DMSO. Error bars represent SEM of one experiment from technical duplicates. Right graph shows cytotoxicity of compounds #69, #79, #87, #93, #96, #102, #103 and remdesivir on their own, normalized to DMSO. Error bars represents SEM of one experiment from technical triplicates. Compounds were applied in a 2-fold serial dilution across 9 concentrations. Starting drug concentrations for each compound were the following: #69: 12 μM, #79: 60 μM, #87: 60 μM, #93: 60 μM, #96: 15 μM, #102: 25 μM, #103: 3 μM and remdesivir: 5 μM. **b**, IF images show anti-N staining (red) and DAPI signal (blue) of the indicated compounds for the graphs above. Last well in each row is the DMSO control.

**Supplementary Figure 9 Synergistic effect of select compounds with remdesivir using SARS-CoV-2 viral infection assay.**

**a**, Representative IF images showing anti-N staining (red) and DAPI signal (blue) of the experiment shown in Figure 4. **b**, Graphs show two additional representative experiments of the antiviral activity (full symbols) and cytotoxicity (empty symbols) of compounds #79, #96 and #102 in A549^+ACE2^ cells infected with SARS-CoV-2 (done in a technical duplicate). Compounds were applied at the following concentrations: #79: 60 μM, #96: 15 μM, #102: 25 μM. Remdesivir concentrations are indicated on the X-axis in μM. Error bars represents SEM. **c**, Schematics showing the overall strategy of our approach.

**SUPPLEMENTARY TABLES**

| Oligo A | 5’6FAM-UUGCCGAAUUAAGCGCA-3’ |
| --- | --- |
|  | 5’BHQ1-UAAGCGCUUAAUUCGGC-3’ |
| Oligo B | 5’6FAM-UUGCCGAAUUAAGCGCCA-3’ |
|  | 5’-UAAGGCGCUUAAUUCGGCAA-3’BHQ1 |
| Oligo C | 5’6FAM-UUUUUUCGGCCCA-3’ |
|  | 5’-AAAUAGGGCCGAAAAAA-3’BHQ1 |
| Oligo D | 5’6FAM-UCUUUUCGGCCCA-3’ |
|  | 5’-AAAUAGGGCCGAAAAGA-3’BHQ1 |
| Oligo E | 5’TxRed-UCUUUUCGGCCCA-3’ |
|  | 5’-AAAUAGGGCCGAAAAGA-3’BHQ2 |
| Oligo B – gel assay | 5’6FAM-UUGCCGAAUUAAGCGCCA-3’ |
|  | 5’-UAAGGCGCUUAAUUCGGCAA-3’ |

**Table S1**

The sequence of the oligonucleotides used in the study.

|  |  | IC50 (μM) |  |
| --- | --- | --- | --- |
|  | SARS-CoV-2 | SARS-CoV | MERS-CoV |
| 69 | 6.65 ± 3.40 | 0.71 | 3.95 |
| 79 | 19.43 ± 8.37 | 4.22 | 4.39 |
| 87 | 10.06 ± 2.40 | 1.22 | 10.23 |
| 93 | 2.20 ± 1.10 | 1.29 | 2.92 |
| 96 | 17.43 ± 1.39 | 14.95 | 21.78 |
| 102 | 21.99 ± 6.02 | 10.08 | 53.26 |
| 103 | 4.12 ± 3.25 | 0.25 | 1.29 |

**Table S2**

The IC_50_ values of compounds #69, #79, #87, #93, #96, #102, #103 for SARS-CoV-2, SARS-CoV and MERS-CoV NSP14 ExoN activity. Error represents SD.
