# Supplementary figures and images for: The NSP14/NSP10 RNA repair complex as a Pan-coronavirus therapeutic target
Source: Cell Death Differ. 2021 Dec 3;29(2):285–92. doi: 10.1038/s41418-021-00900-1 (PMC8640510; doi:10.1038/s41418-021-00900-1)

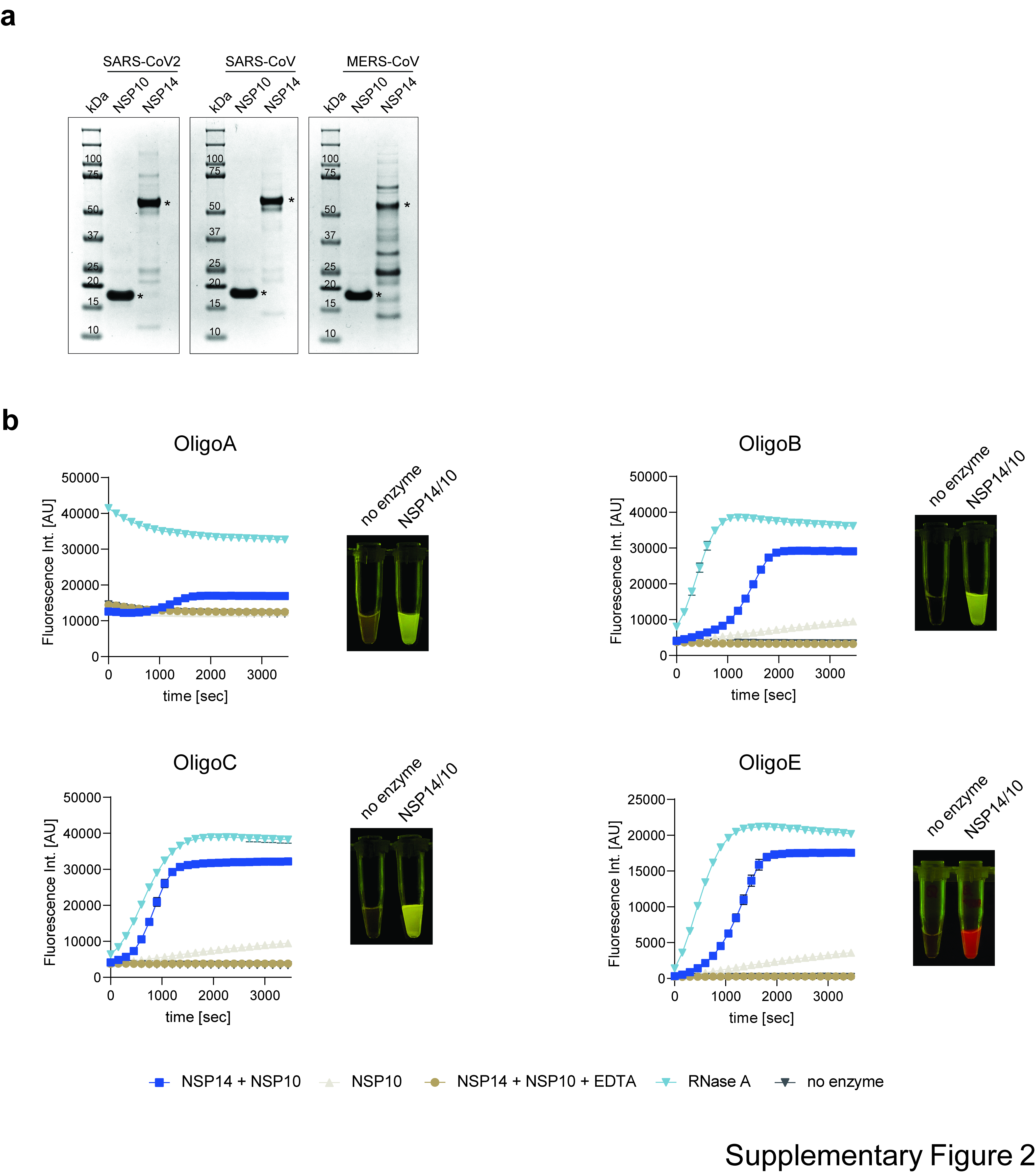

Supplement: Supplementary file 3 — Supplementary Figure 2 In vitro FRET-based NSP14 activity assay optimization and characterization. [file 41418_2021_900_MOESM3_ESM.tif]

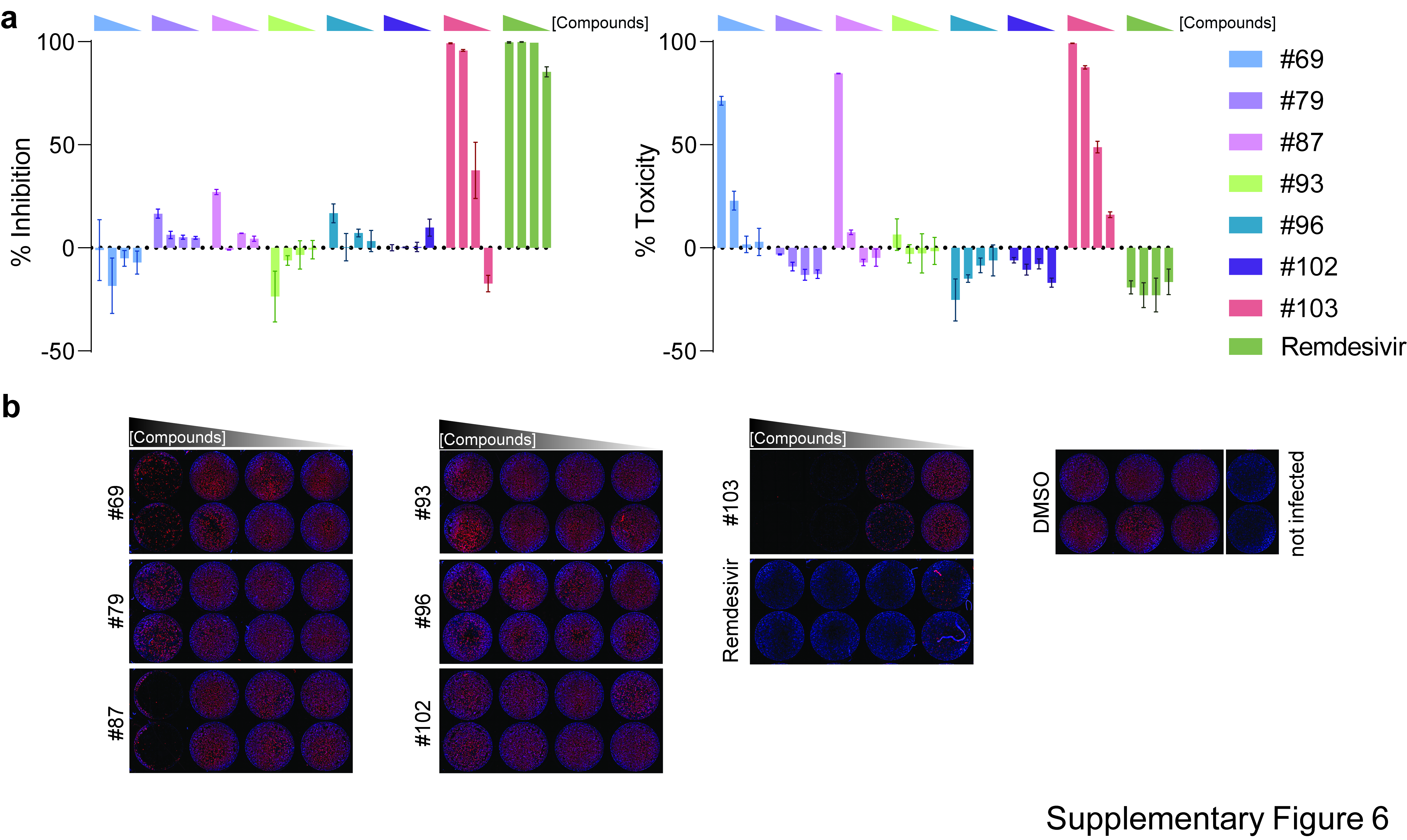

Supplement: Supplementary file 7 — Supplementary Figure 6 Viral infection assay using HCoV-OC43. [file 41418_2021_900_MOESM7_ESM.tif]

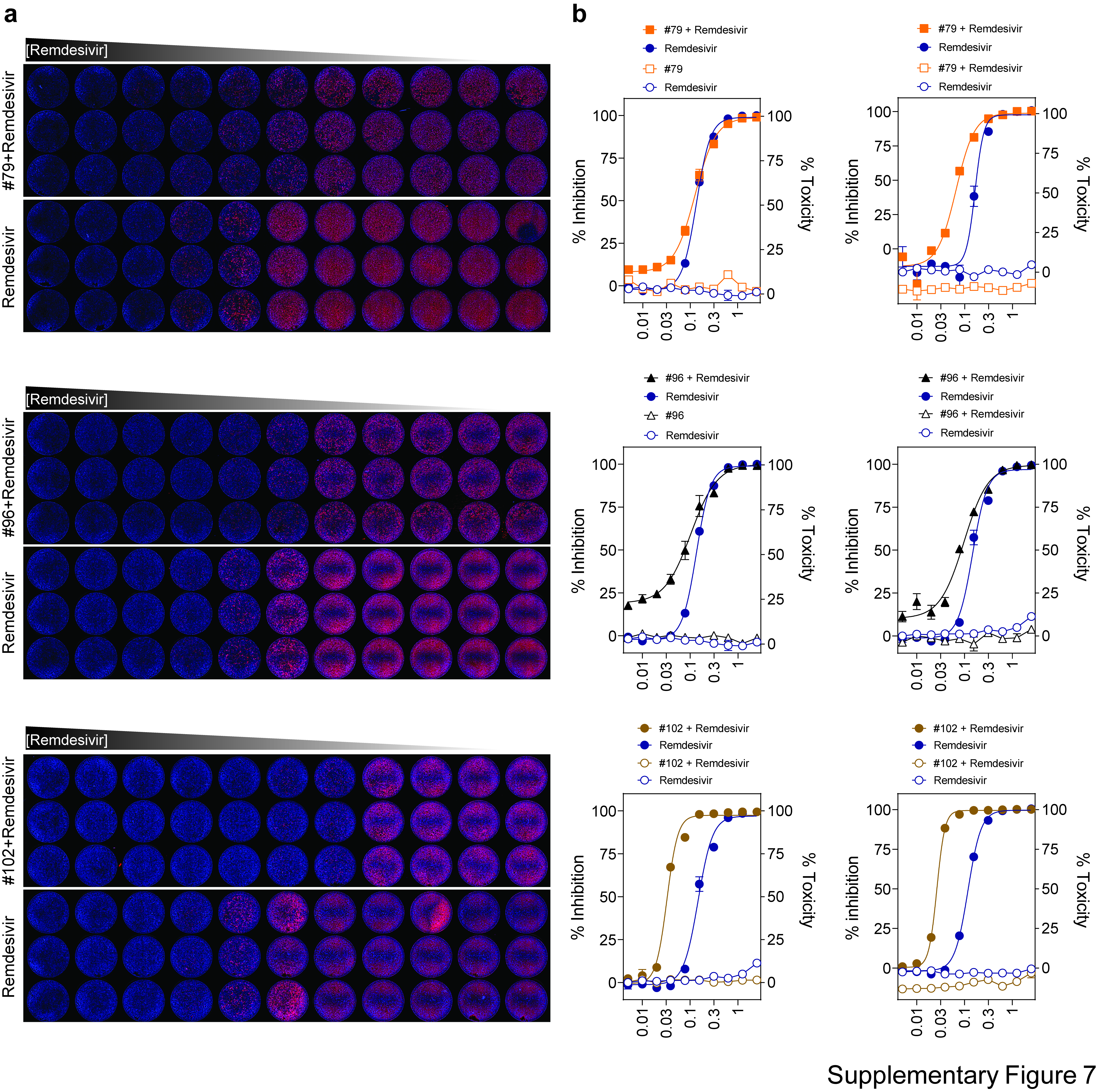

Supplement: Supplementary file 8 — Supplementary Figure 7 Synergistic effect of select compounds with remdesivir using HCoV-OC43 viral infection assay. [file 41418_2021_900_MOESM8_ESM.tif]

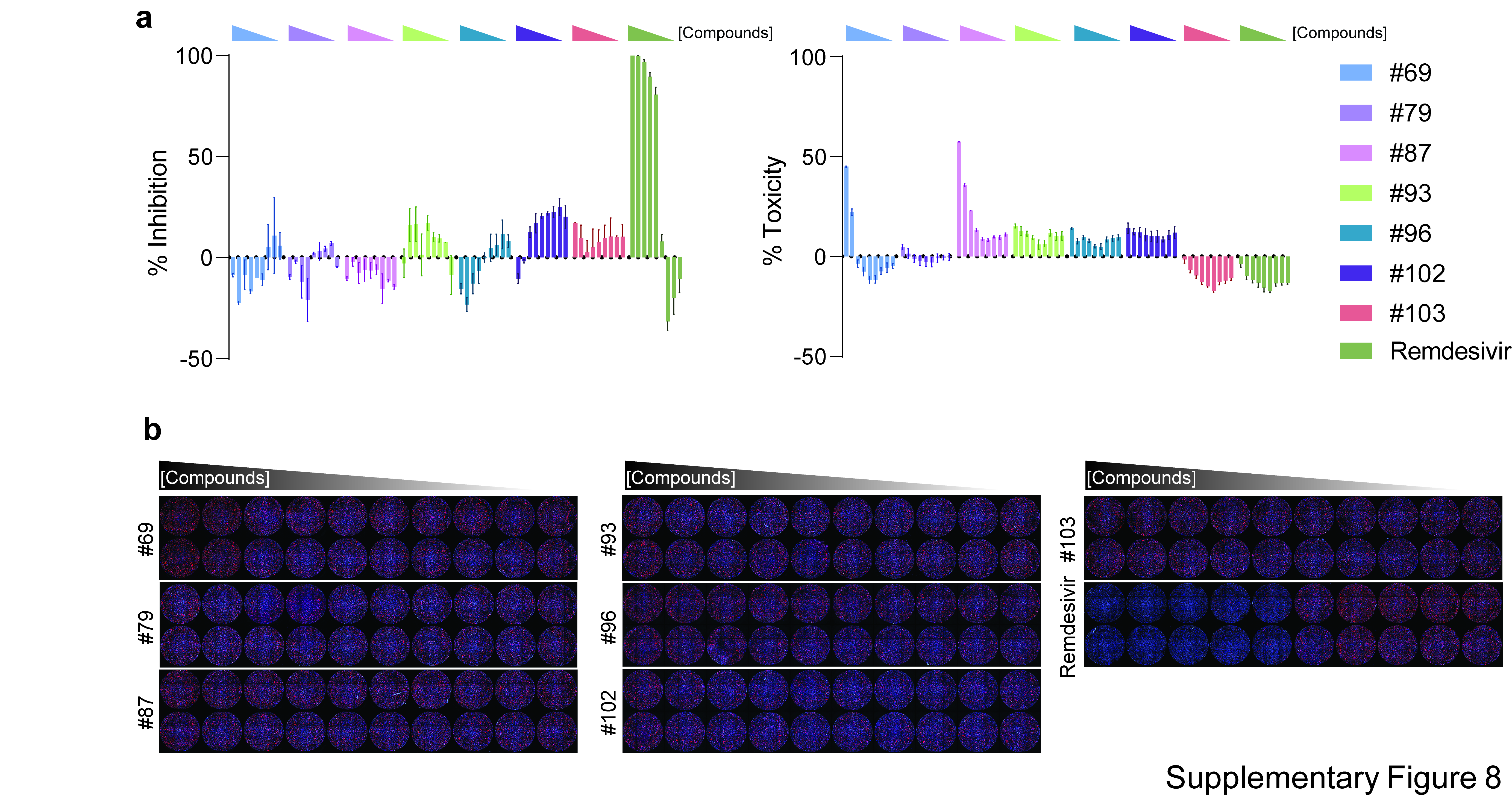

Supplement: Supplementary file 9 — Supplementary Figure 8 Viral infection assay using SARS-CoV-2. [file 41418_2021_900_MOESM9_ESM.tif]
