# Supplementary material for: The NSP14/NSP10 RNA repair complex as a Pan-coronavirus therapeutic target
Source: Cell Death Differ. 2021 Dec 3;29(2):285–92. doi: 10.1038/s41418-021-00900-1 (PMC8640510; doi:10.1038/s41418-021-00900-1)
Supplement: Supplementary file 12 — Detailed Author Contribution Form [file 41418_2021_900_MOESM12_ESM.pdf]

DECLARATION OF CONTRIBUTIONS TO ARTICLE

ADMC

Manuscript Number: CDD-21-1935RR

Journal Name: *Cell Death & Differentiation* (the 'Journal')

Proposed Title of the Contribution:

The NSP14/NSP10 RNA Repair Complex as a Pan-coronavirus Therapeutic Target (the 'Contribution)

Author(s):

Gergely Rona, Andras Zeke, Bearach Miwatani-Minter, Maren de Vries, Ramanjit Kaur, Austin Schinlever, Sheena Faye Garcia, Hailey V. Goldberg, Hui Wang, Thomas R. Hinds, Fabrice Bailly, Ning Zheng, Philippe Cotelle, Didier Desmaële, Nathaniel R. Landau, Meike Dittmann, Michele Pagano (the 'Authors')

For all *CDD* articles, each person named as an author in the published version must be able to show he or she has contributed substantially to the article.

Authorship credit should be based on 1) substantial contributions to conception and design, acquisition of data, or analysis and interpretation of data; 2) drafting the article or revising it critically for important intellectual content; and 3) final approval of the version to be published. Authors should meet conditions 1, 2 and 3.

Any person who cannot be shown to have made a substantial contribution to the article cannot be listed as an author in the final version. The name of any person who is deemed to have made a minor contribution can, however, appear in the Acknowledgments section of the article.

Please complete the table below to indicate the contributions of all named authors to the manuscript.

Author Full Name:	Specification of Contribution to the Manuscript:
Gergely Rona, Andras Zeke, Bearach Miwatani-Minter	Conceived, planned, and performed most experiments, and co-wrote the manuscript.
Maren de Vries, Ramanjit Kaur, Austin Schinlever, Nathaniel R. Landau	Performed and/or provided help with the viral infection assays.
Sheena Faye Garcia, Hailey V. Goldberg	Helped with and performed protein purification.
Hui Wang, Thomas R. Hinds, Ning Zheng	Advised about some biochemical experiments.
Fabrice Bailly, Philippe Cotelle, Didier Desmaële	Synthesized Styrylquinoline and NHID compounds.
Meike Dittmann	Directed and coordinated the BSL3 studies.
Michele Pagano	Conceived, directed, and coordinated the study, oversaw all the results, and co-wrote the manuscript.
All authors	All authors discussed the results and commented on the manuscript.

Please complete the table below to indicate the contributions of all named authors to the figures.

Figure 1:

All the authors.

Figure 2:

All the authors.

Figure 3:

All the authors.

Figure 4:

All the authors.

Figure 5:

All the authors.

Figure 6:

Signed for and on behalf of the Author(s):	Print Name:	Date:
	Michele Pagano	11/08/2021
